# Dietary and economic effects of eliminating shortfall in fruit intake on nutrient intakes and diet cost

**DOI:** 10.1186/s12887-016-0620-z

**Published:** 2016-07-07

**Authors:** Colin D. Rehm, Adam Drewnowski

**Affiliations:** Department of Epidemiology and Center for Public Health Nutrition, University of Washington, Box 353410, Seattle, WA 98195 USA; Institute for Cardiometabolism and Nutrition (ICAN), Université Pierre et Marie Curie - Paris VI, Groupe Hospitalier Pitié-Salpêtrière, 91 boulevard de l’Hôpital, 75013 Paris, France

**Keywords:** Diet, Nutritional status, Fruit, Child nutrition sciences, Nutrition policy

## Abstract

**Background:**

Children in the United States do not consume the recommended amounts of fruit. The economic and dietary consequences of meeting the shortfall in fruit consumption have not been evaluated.

**Methods:**

Analyses were based on a nationally representative sample of 4–18 year-old children (*n* = 2,647) from the 2009–2010 National Health and Nutrition Examination Survey (NHANES). The shortfall in total fruit consumption for each child was estimated based on the USDA MyPlate recommendations. The potential impact of filling the shortfall in total fruit consumption was projected with whole fruit alone (WF model) or a combination of 100 % fruit juice and whole fruit (FJ + WF model). Juice consumption was capped using American Academy of Pediatrics (AAP) standards. The USDA national food prices database was used to estimate the cost of meeting the dietary recommendations for fruit. Selected nutrient and mineral intakes, as well as daily diet cost were estimated after eliminating the shortfall in fruit consumption.

**Results:**

Among all children, vitamin C (+22.8 mg [95 % CI 21.4, 24.1] in the WF model and +48.1 mg [95 % CI 45.2, 51.1] in the FJ + WF model) and potassium intakes (+203 mg [95 % CI 190, 215] in WF and +263 mg [95 % CI 248, 280] in FJ + WF) were increased in both models. The FJ + WF model resulted in a marginal increase in dietary fiber (e.g., a relative change less than 10 %), while the WF model resulted in a meaningful increase in dietary fiber (e.g., a relative change greater than 10 %; +2.2 g [95 % CI 2.1, 2.3]). Conversely, the WF model resulted in only a marginal increase in calcium, while the FJ + WF model resulted in a meaningful increase in calcium (+85 mg [95 % CI 79, 89]). Calories were increased in all models (+4.5 % [95 % CI 4.1, 4.9 %] for FJ + WF and +3.5 % [95 % CI 3.2, 3.7 %] for WF). Meeting the fruit shortfall with whole fruit alone increased estimated diet costs by 9.9 % (+$0.44/d [95 % CI 0.42, 0.47]), while the fruit juice/whole fruit combination increased diet costs by 5.2 % (+$0.23/d [95 % CI 0.22, 0.25]).

**Conclusions:**

Meeting fruit consumption guidelines without a substantial increase in diet costs may be a challenge. Combining whole fruit with 100 % fruit juice capped at AAP standards may be one approach to meeting fruit recommendations within cost constraints. Identifying approaches to increasing whole fruit consumption in as cost-neutral a fashion as possible should be a priority.

## Background

Dietary intakes of fruit by US children fall far short of national recommendations [1, 2]. The Choose MyPlate recommends consuming between 1.0 to 2.5 cups of fruit per day, depending on age, gender, and physical activity [3]. By contrast, the estimated mean daily intakes for fruit (including 100 % fruit juice) for 4–18y olds are about 1 cup per day, though older children consume less [4]. For reference, 1.0 cup of fruit corresponds to a small apple (106 g), large banana (136 g), 32 seedless grapes (160 g), large orange (184 g), 8 large strawberries (166 g) or 1 cup of fruit juice (248 g or 8 fluid ounces). Numerous interventions, in pre-school [5], school [6], and family settings [7] have been developed to address the shortfall and fruit consumption and to improve the quality of children’s diets, though fruit consumption remains well below recommended levels. Consuming adequate amounts of fruit is important in ensuring nutrient adequacy, particularly for vitamin C, potassium, and dietary fiber, and may also reduce the risk of weight gain due to their low energy density [1, 2]. Furthermore, fruit consumption in childhood predicts fruit consumption in adulthood, which may reduce the risk of some chronic diseases, including cardiovascular disease [8, 9].

Approximately, 40 % of total fruit consumed by children comes from 100 % fruit juice [4]. The consumption of 100 % fruit juice, in observational studies, has been found to be associated with an increased risk of childhood obesity, and some prospective studies among adults have observed that juice consumption is associated with an increased risk of diabetes [10–12]. One argument is that sugar consumption in the absence of dietary fiber is associated with excess weight gain, liver injury and the metabolic syndrome [10]. To this end, the American Academy of Pediatrics (AAP) recommends 100 % fruit juice be limited to 4–6 fl oz for children aged 1–6y and 8–12 fl oz for children 7–18y [13]. The American Heart Association recommends no more than 4–6 fl oz of fruit juice per day and the new food package for the Women, Infants and Children (WIC) program reduced 100 % juice allowances by half [14].

The consumption of total fruit by children in the United States follows a socioeconomic gradient [4, 6, 15, 16]. In a study based on 1999–2002 NHANES data, fruit consumption among children was associated with living in a food secure and higher income household [4]. In modeled systems, replacing energy dense foods with vegetables and fruit led to a sharp increase in diet costs [17]. Observational studies also show that the diets of individuals consuming higher cost diets typically contain more total fruit than diets of individuals consuming lower cost diets, with the disparity being particularly apparent for whole fruits [18, 19]. On a per-calorie basis among adults, more costly diets contained significantly more whole fruit than lower cost diets [18, 19]. The high cost of whole fruits on a per-calorie basis is one possible factor explaining the socioeconomic gradient in whole fruit consumption [20, 21].

Therefore, increasing children’s fruit intake represents an economic and a behavioral challenge. While behavioral/educational interventions have been discussed in detail [22–25], there are limited studies on the nutritional and economic impact of replacing juice with fruit or vice versa [26]. The present study explored the economic and nutritional impact of reaching the dietary recommendations for fruit using either whole fruit or a combination of fruit and 100 % juice, using amounts in line with AAP recommendations. The nutritional outcomes of interest were total energy intake, vitamin C, potassium, calcium and dietary fiber.

## Methods

### NHANES participants & dietary assessment

Data analyses were based on the National Health and Nutrition Examination Survey from 2009–2010. Data for 2,647 children 4–18y were available for analysis.

The NHANES 24-h dietary recall utilized a multi-pass method, where respondents reported the types and amounts of all food and beverages consumed in the preceding 24-h. For children 4–5y, the dietary recall was completed entirely by a parent/guardian. For children 6–11y, the child was the primary respondent, but a proxy respondent was present and able to assist. For 12–18y olds, the child was the primary respondent, but could be assisted by an adult.

The NHANES database includes two dietary recalls for most participants. The first was completed in-person at the Mobile Examination Center with an interviewer. The second was completed over the telephone and tends to result in lower estimated energy intakes than in-person [27]. Because the goal of this project was to assess the nutritional and economic consequences of meeting fruit recommendations under different approaches, the first 24-h recall was used for all analyses and is adequate to evaluate average population-level dietary intakes [28].

To address the shortfall in fruit consumption, current consumption of total fruit, whole fruit, and 100 % fruit juice, expressed in terms of cup equivalents, were estimated based on USDA data. A cup equivalent of fruit corresponds to 1 whole fruit, a cup of sliced fruit, ½ cup of dried fruit or 1 cup of 100 % juice (8 fl oz) [29]. For example, 1.0 cup of fruit corresponds to a small apple (106 g), large banana (136 g), 32 seedless grapes (160 g), large orange (184 g), 8 large strawberries (166 g) or 1 cup of fruit juice (248 g or 8 fluid ounces). Cup equivalents for fruit were obtained from the USDA MyPyramid Equivalents Database (MPED). Because updated MPED data were not available for the 2009–2010 NHANES data, we used the MPED addendum database from the Center for Policy and Promotion (CNPP). Analyses were conducted prior to the September 2013 release of the Food Patterns Equivalent Database, containing updated groups related to 2010 Dietary Guidelines. Whole fruit and fruit juices were differentiated using the 2003–2004 Center for Nutrition Policy and Promotion (CNPP) Healthy Eating Index support files, which includes information on the relative content of whole vs. fruit juice per 100 g for each food/beverage.

### The national food prices database

The USDA CNPP national food prices database includes average national prices for more than 6000 foods and beverages that were consumed by NHANES respondents in 2003–2004. To evaluate the economic dimensions of fruit and fruit juice consumption in the more recent NHANES data, the CNPP food prices database was adjusted for inflation. To adjust food prices, first, all foods from 2009–2010 NHANES missing a 2003–2004 price were identified and a 2003–2004 price was estimated using similar foods. The 2003–2004 prices were then adjusted for inflation and price increases using food-group specific adjustment factors as food prices have not changed uniformly over time [30, 31]. Food group definitions were obtained form a prior USDA study assessing food sources of sodium based on NHANES data [32], which was linked to the USDA Quarterly Food At-Home Price Database (QFAHPD) [33]. The QFAHPD includes average prices by quarter from 2004–2010 for specific food groups across multiple markets [33]. The QFAHPD included 54 food groups and the USDA food groups included 104 food groups. For example: from the USDA food categories “milk, lowfat and nonfat” was linked with “low fat milk” from the QFAHPD.

For food groups with multiple matches within the QFAHPD, price values were distributed between multiple components of a given food group, based on weighted frequency of consumption of items within each food group. For example, for “apples”, we used the prices for “fresh/frozen fruit” and “canned fruit”, but weighted them 0.915 and 0.085 respectively, based on the relative frequency of consumption of raw apples and applesauce. This approach weighted the quarterly prices equally, not accounting for the seasonal availability of some items. Because the QFAHPD is based on a weighted sample we accounted for the survey weights so the food group specific adjustment factors represent the change in price at the national level. The prices database represents the average retail price at the national level and assumes all food items were purchased from stores, not restaurants.

### Models to address fruit shortfall

The shortfall in total fruit intake for each participant was quantified by identifying optimum amounts of total fruit by age and gender group based on the Choose MyPlate recommendations [3]. For children 4–5y and girls 6–8y, 1.0 cup was the reference value. For boys 6–8y, all children 9–12y, and girls 13–18y, 1.5 cups was the reference value. For boys 13–18y, 2 servings was the reference value. The shortfall was defined as the difference between the reference value and the amount consumed by each participant assuming that the participant consumed less than the reference amount.

Models were then developed to obtain the recommended amount of total fruit. In the Fruit Juice plus Whole Fruit (FJ + WF) Model, dietary recommendations for fruit were reached using a combination of 100 % fruit juice and whole fruit, though the amount of 100 % fruit juice that could be used to achieve the recommended fruit intake was capped at the level recommended by the American Academy of Pediatrics (1.5 servings of fruit juice for age 8+ and 0.63 for age 4–7) and whole fruit was used thereafter [13]. The limit applied both to fruit juice consumed and juice included in the modeling. An individual consuming more than 1.5 servings of whole fruit would only have up-to 1.5 servings of 100 % fruit juice used in the models. While individuals already consuming more than 1.5 servings of fruit juice (or 0.63 for young children) were included in the analysis, in no case did the modeling increase the number of fruit juice servings beyond the recommended maximum number of servings. An alternative Whole Fruit (WF) Model was specified that met dietary recommendations for total fruit using whole fruit alone, including fresh, frozen, canned and dried varieties, which were weighted by their frequency of consumption.

Rather than selecting arbitrary whole fruits and fruit juices for modeling, a composite whole fruit and fruit juice was created based on a weighted estimate using their relative frequency of consumption. For whole fruits, all fresh, canned, frozen and dried fruit that were not part of mixed dishes and consumed by children 4–18y were flagged. For fruit juices, all 100 % fruit juices were flagged. The number of times each item was reported was used to derive a weight for the composite fruit (e.g., fresh apples were consumed 426 times and had a weight of 0.223). The most important whole fruits were apples (weight = 0.223), bananas (0.145), oranges (0.097), grapes (0.095), and strawberries (0.067). While canned, frozen and dried fruit are lower-cost fruit sources, they were infrequently consumed (e.g., their collective weight was 0.12). The fruit juices most heavily weighted in the composite fruit juice were orange juice without calcium (weight = 0.270), orange juice with calcium (0.196), apple juice (0.191), fruit juice blends (0.153), and grape juice (0.051).

### Analytical approach

The outcomes of interest were important vitamins and minerals from whole fruit and 100 % fruit juice, including vitamin C, dietary fiber, calcium, and potassium. Fiber, calcium and potassium were all identified in the 2010 Dietary Guidelines for Americans as nutrients of concern [2]. Vitamin C was selected because fruit is an important source of this nutrient [2]. Estimated daily diet cost was an additional outcome. The survey-weighted mean value for the nutrients and diet cost measure was calculated as observed and based on the models. Data are presented for the entire population and for those children with a fruit shortfall for all children/adolescents and stratified by age group (4–8y, 9–13y and 14–18y). Additional stratified analyses were conducted by race/ethnicity and family income. For models conducted with the shortfall population only, the diets of children consuming adequate amounts of fruit was not altered.

By the nature of the models, energy, micronutrient/mineral, and diet costs were all statistically greater after removing the fruit shortfall (i.e., because the amount of food consumed requisitely increased). Because of this, *p*-values comparing the observed mean to the modeled mean are not estimated, rather we present data on the relative and absolute changes along with corresponding 95 % confidence intervals (CI). All analyses accounted for the complex survey design of NHANES data using Stata 13 (StataCorp, College Station, TX). Because the nature of the models resulted in highly statistically significant changes in the dietary outcomes being evaluated, qualitative comparisons were considered based on the percent change from the observed diets. A 10 % change was considered qualitatively meaningful, while a change of less than 10 % was considered to be marginal or modest.

### Ethical approval and availability of supporting data

Publicly available data, such as those from NHANES, are considered exempt from Human Subjects review per University of Washington policies. All data used in this research are available to the public at the websites for the National Center for Health Statistics and the Agriculture Research Service of the United States Department of Agriculture.

## Results

Table 1 shows mean servings of total fruit, whole fruit and 100 % fruit juice, as well as estimated daily diet cost by age group, gender, income to poverty ratio and race/ethnicity. Overall, children and adolescents consumed 1.1 (95 % CI 1.0, 1.2) servings of total fruit per day, 59 % from whole fruit and the remainder from fruit juice. Most children (68.8 %) had a shortfall in fruit consumption. Fruit consumption did not differ substantially by gender. Younger children consumed significantly more total fruit and whole fruit than older children. Differences in total fruit intake were not observed by family income, though children from lower-income families received a greater proportion of their total fruit from fruit juice than those from higher-income families. Total fruit intake did differ by race/ethnicity. Mexican-American children consumed the most total fruit and non-Hispanic black children consumed the most fruit juice. Fifty-two percent of total fruit among non-Hispanic black children came from fruit juice compared to 34 % for non-Hispanic white children.

**Table 1:** Mean servings of total fruit, whole fruit, and 100 % fruit juice and estimated diet cost by age group, gender, family income-to-poverty ratio, and race/ethnicity among children and adolescents age 4–18y, NHANES 2009–2010 (*n* = 2,647)

	n	Total fruit^a^	Whole fruit	Fruit juice	Estimated diet cost ($)
		(95 % CI)	(95 % CI)	(95 % CI)	(95 % CI)
Total	2,647	1.11 (1.00, 1.22)	0.66 (0.58, 0.74)	0.44 (0.38, 0.51)	4.48 (4.28, 4.69)
Gender					
Female (ref)	1,282	1.05 (0.94, 1.17)	0.64 (0.55, 0.73)	0.41 (0.34, 0.48)	4.12 (3.95, 4.29)
Male	1,365	1.16 (1.02, 1.30)	0.68 (0.58, 0.78)	0.48 (0.39, 0.57)	4.85 (4.56, 5.14)^#^
Age group (y)					
4–8	958	1.29 (1.16, 1.43)^¶^	0.78 (0.68, 0.88)^*^	0.51 (0.44, 0.59)	3.83 (3.73, 3.93)^#^
9–13	887	1.00 (0.89, 1.11)	0.62 (0.56, 0.68)	0.38 (0.29, 0.47)	4.42 (4.30, 4.54)^#^
14–18 (ref)	802	1.03 (0.84, 1.22)	0.59 (0.41, 0.76)	0.44 (0.33, 0.55)	5.15 (4.68, 5.62)
Income-to-poverty ratio					
< 1.3	1,122	1.08 (0.94, 1.22)	0.59 (0.52, 0.66)	0.49 (0.38, 0.59)	4.31 (4.10, 4.51)
1.3–2.99	696	1.11 (0.79, 1.43)	0.63 (0.37, 0.9)	0.48 (0.37, 0.59)	4.22 (3.95, 4.49)^*^
≥ 3.00 (ref)	606	1.07 (0.96, 1.18)	0.71 (0.59, 0.83)	0.36 (0.29, 0.43)	4.75 (4.33, 5.17)
Race/ethnicity					
Mexican-American	746	1.34 (1.06, 1.61)^*^	0.75 (0.63, 0.88)	0.59 (0.41, 0.77)^*^	4.23 (4.02, 4.44)^*^
Other Hispanic	307	1.19 (0.98, 1.40)	0.67 (0.49, 0.85)	0.52 (0.44, 0.60)^¶^	4.16 (3.95, 4.37)^*^
Non-Hispanic white (ref)	883	1.00 (0.85, 1.14)	0.65 (0.52, 0.79)	0.34 (0.28, 0.40)	4.58 (4.28, 4.88)
Non-Hispanic black	525	1.18 (1.01, 1.36)	0.58 (0.49, 0.67)	0.61 (0.47, 0.74)^#^	4.37 (4.07, 4.68)

Reference group identified in parentheses. Statistical significance is indicated as follows; ^*^ 0.01 < *p*-value < 0.05; ^¶^ 0.001 < *p*-value < 0.01; ^#^ *p*-value < 0.001

^a^A serving size of fruit corresponds to a cup equivalent. One cup equivalent of fruit corresponds to a small apple (106 g), large banana (136 g), 32 seedless grapes (160 g), large orange (184 g), 8 large strawberries (166 g) or 1 cup of fruit juice (248 g or 8 fluid ounces)

The results of modeling to meet the total fruit shortfall among all children (*n* = 2,647) and children with a shortfall in fruit consumption (*n* = 1,781) are shown in Table 2 and Table 3, respectively. Data are presented for all children and by age group. Among all children and those with a shortfall in fruit intake, the average shortfall was 0.75 (95 % CI 0.71, 0.80) and 1.09 servings (95 % CI 1.06, 1.12), respectively. First, meeting total fruit guidelines by whole fruit (WF) alone or a combination of fruit juice and whole fruit (FJ + WF) would increase total energy intakes by no more than 6 % or less than 100 kcal/d, with the WF + FJ model resulting in greater increases in total energy than the WF model. In the FJ + WF model, vitamin C, dietary fiber, calcium and potassium increased by +61 % (+48 mg/d), +4.7 % (+0.7 g/d), +7.9 % (+85 mg/d), and +11.7 % (264 mg/d) respectively. In the WF model vitamin C, dietary fiber, calcium and potassium increased by +29 % (23 mg/d), +16 % (+2.2 g/d), +1.3 % (+14 mg/d), and +9.0 % (+203 mg/d), respectively. When comparing the impacts of the two models to each other, the FJ + WF model resulted in significant increases in vitamin C (+25.1 % [95 % CI 22.2, 27.9], comparing WF + FJ to the FJ model), and modest increases in calcium (6.5 % [95 % CI 5.9, 7.1) and potassium (2.5 % [95 % CI 2.3, 2.7]), while the WF model resulted in 9.5 % (95 % CI 8.7, 10.3) lower dietary fiber. The FJ + WF model resulted in a 5.2 % (95 % CI 4.7, 5.7 %) increase in diet costs, while the WF model resulted in a 9.9 % increase (95 % CI 9.0, 10.8 %). Comparing the two models to each other, the WF + FJ model resulted in a 4.3 % (95 % CI 3.9, 4.6) decrease in diet cost when compared to the WF model. In general, results were similar across age groups (shown in Table 2) and when stratified by race/ethnicity and family income (data not shown).

**Table 2:** Observed and model predicted total energy intake (kcal), nutrient intakes, and estimated diet cost among all children and adolescents age 4–18, NHANES 2009–2010

	Observed	Fruit juice + whole fruit (FJ + WF) model			Whole fruit (WF) model			Comparison of FJ + WF vs. WF mean	
	Mean (95 % CI)	Mean (95 % CI)	Absolute change from observed (95 % CI)	% change from observed (95 % CI)	Mean (95 % CI)	Absolute change from observed (95 % CI)	% change from observed (95 % CI)	Absolute change from FJ + WF vs. WF Model (95 % CI)	% change from FJ + WF vs. WF model (95 % CI)
Energy (kcal)									
Overall^a^	1969 (1908, 2030)	2058 (1999, 2116)	88 (83, 94)	4.5 (4.1, 4.9)	2037 (1978, 2096)	68 (64, 72)	3.5 (3.2, 3.7)	20 (19, 22)	1.0 (0.9, 1.1)
Age 4–8y^b^	1740 (1688, 1791)	1786 (1737, 1836)	47 (40, 53)	2.7 (2.3, 3.1)	1777 (1727, 1827)	37 (32, 43)	2.2 (1.8, 2.5)	9 (8, 11)	0.5 (0.4, 0.6)
Age 9–13y^c^	1937 (1868, 2006)	2032 (1964, 2100)	95 (87, 103)	4.9 (4.4, 5.4)	2009 (1941, 2077)	72 (66, 78)	3.7 (3.3, 4.1)	23 (21, 25)	1.2 (1.0, 1.3)
Age 14–18y^d^	2216 (2088, 2344)	2338 (2213, 2462)	122 (115, 129)	5.5 (5.0, 6.0)	2310 (2184, 2435)	94 (88, 99)	4.2 (3.8, 4.6)	28 (26, 30)	1.2 (1.1, 1.3)
Vitamin C (mg)									
Overall	78 (71, 86)	127 (122, 132)	48 (45, 51)	61.4 (52.5, 70.3)	101 (95, 107)	23 (21, 24)	29.1 (24.9, 33.2)	25 (24, 27)	25.1 (22.2, 27.9)
Age 4–8y	79 (71, 86)	103 (97, 108)	24 (21, 28)	30.8 (23.7, 37.9)	91 (84, 98)	13 (11, 14)	15.9 (12.4, 19.5)	12 (10, 13)	12.8 (10.1, 15.5)
Age 9–13y	71 (61, 81)	124 (115, 132)	53 (49, 57)	74.5 (60.3, 88.7)	95 (86, 104)	24 (22, 26)	33.9 (27.4, 40.3)	29 (27, 31)	30.4 (26.1, 34.7)
Age 14–18y	85 (73, 98)	152 (141, 162)	66 (62, 70)	77.8 (62.5, 93.1)	117 (105, 128)	31 (30, 33)	36.7 (29.6, 43.9)	35 (33, 37)	30 (25.7, 34.4)
Dietary fiber (gm)									
Overall	13.9 (13.4, 14.5)	14.6 (14, 15.2)	0.7 (0.6, 0.7)	4.7 (4.2, 5.1)	16.1 (15.6, 16.6)	2.2 (2.1, 2.3)	15.7 (14.2, 17.2)	−1.5 (−1.6, −1.4)	−9.5 (−10.3, −8.7)
Age 4–8y	12.9 (12.4, 13.4)	13.4 (12.9, 13.9)	0.5 (0.4, 0.6)	3.8 (3.2, 4.5)	14.1 (13.7, 14.6)	1.2 (1.0, 1.4)	9.3 (7.8, 10.8)	−0.7 (−0.8, −0.6)	−5.0 (−5.8, −4.2)
Age 9–13y	14.3 (13.3, 15.3)	14.9 (13.9, 15.8)	0.6 (0.5, 0.6)	3.9 (3.4, 4.5)	16.6 (15.7, 17.5)	2.3 (2.1, 2.5)	16.1 (14.1, 18.1)	−1.7 (−1.9, −1.6)	−10.5 (−11.7, −9.4)
Age 14–18y	14.6 (13.7, 15.5)	15.5 (14.6, 16.3)	0.9 (0.8, 0.9)	6.1 (5.5, 6.7)	17.6 (16.9, 18.4)	3.0 (2.8, 3.2)	20.6 (18.5, 22.8)	−2.1 (−2.3, −2.0)	−12.1 (−13.2, −10.9)
Calcium (mg)									
Overall	1068 (1022, 1113)	1152 (1110, 1194)	85 (79, 90)	7.9 (7.2, 8.7)	1082 (1037, 1127)	14 (13, 15)	1.3 (1.2, 1.5)	70 (66, 75)	6.5 (5.9, 7.1)
Age 4–8y	1027 (973, 1080)	1067 (1014, 1121)	40 (34, 46)	3.9 (3.3, 4.5)	1035 (981, 1088)	8 (7, 9)	0.8 (0.6, 0.9)	32 (28, 37)	3.1 (2.6, 3.6)
Age 9–13y	1061 (986, 1136)	1156 (1081, 1230)	95 (87, 103)	9.0 (7.9, 10.0)	1076 (1001, 1151)	15 (14, 16)	1.4 (1.3, 1.6)	80 (73, 86)	7.4 (6.6, 8.3)
Age 14–18y	1132 (1053, 1211)	1132 (1053, 1211)	117 (110, 124)	10.5 (9.2, 11.8)	1132 (1053, 1211)	20 (19, 21)	1.8 (1.6, 2.0)	97 (91, 103)	8.6 (7.5, 9.6)
Potassium (mg)									
Overall	2244 (2156, 2333)	2508 (2430, 2586)	264 (248, 280)	11.7 (10.7, 12.8)	2447 (2367, 2527)	203 (191, 215)	9.0 (8.2, 9.9)	61 (57, 65)	2.5 (2.3, 2.7)
Age 4–8y	2105 (2021, 2190)	2245 (2172, 2318)	140 (120, 159)	6.6 (5.5, 7.8)	2217 (2142, 2292)	112 (96, 127)	5.3 (4.4, 6.2)	28 (24, 32)	1.3 (1, 1.5)
Age 9–13y	2183 (2067, 2299)	2466 (2356, 2575)	283 (259, 306)	13 (11.5, 14.4)	2396 (2286, 2507)	214 (196, 232)	9.8 (8.7, 10.9)	69 (64, 75)	2.9 (2.6, 3.2)
Age 14–18y	2434 (2266, 2601)	2797 (2644, 2950)	363 (342, 384)	14.9 (13.2, 16.7)	2712 (2556, 2869)	279 (263, 295)	11.5 (10.1, 12.8)	84 (79, 90)	3.1 (2.8, 3.5)
Cost ($/d)									
Overall	4.48 (4.28, 4.69)	4.72 (4.52, 4.91)	0.23 (0.22, 0.25)	5.2 (4.7, 5.7)	4.93 (4.73, 5.12)	0.44 (0.42, 0.47)	9.9 (9.0, 10.8)	−0.21 (−0.22, −0.2)	−4.3 (−4.6, −3.9)
Age 4–8y	3.83 (3.73, 3.93)	3.98 (3.89, 4.07)	0.15 (0.13, 0.17)	3.8 (3.2, 4.4)	4.08 (3.99, 4.17)	0.24 (0.21, 0.28)	6.4 (5.4, 7.3)	−0.1 (–0.11,–0.08)	–2.4 (−2.8, −2.0)
Age 9–13y	4.42 (4.30, 4.54)	4.65 (4.53, 4.77)	0.23 (0.21, 0.25)	5.2 (4.7, 5.7)	4.89 (4.77, 5.02)	0.47 (0.43, 0.51)	10.6 (9.6, 11.5)	−0.24 (−0.26, −0.22)	−4.9 (−5.3, −4.5)
Age 14–18y	5.15 (4.68, 5.62)	5.47 (5.01, 5.93)	0.32 (0.3, 0.34)	6.2 (5.4, 7.0)	5.76 (5.31, 6.22)	0.61 (0.58, 0.65)	11.8 (10.3, 13.4)	−0.29 (−0.31, −0.27)	−5.1 (−5.7, −4.4)

^a^ The mean shortfall in the overall population was 0.75 (95 % CI 0.71, 0.80) and 68.8 % had a shortfall in total fruit intake

^b^ The mean shortfall among those 4–8y was 0.41 (95 % CI 0.36, 0.47) and 53.3 % had a shortfall in total fruit intake

^c^ The mean shortfall among those 9–13y was 0.79 (95 % CI 0.72, 0.86) and 75.0 % had a shortfall in total fruit intake

^d^ The mean shortfall among those 14–18y was 1.03 (95 % CI 0.97, 1.09) and 77.9 % had a shortfall in total fruit intake

**Table 3:** Observed and model predicted total energy intake (kcal), nutrient intakes, and estimated diet cost among children and adolescents age 4–18 with a shortfall in total fruit consumption, NHANES 2009–2010 (*n* = 1,781)

	Observed	Fruit juice + whole fruit (FJ + WF) model			Whole fruit (WF) model			Comparison of FJ + WF vs. WF mean	
	Mean (95 % CI)	Mean (95 % CI)	Absolute change from observed (95 % CI)	% change from observed (95 % CI)	Mean (95 % CI)	Absolute change from observed (95 % CI)	% change from observed (95 % CI)	Absolute change from FJ + WF vs. WF Model (95 % CI)	% change from FJ + WF vs. WF model (95 % CI)
Energy (kcal)									
Overall^a^	1946 (1883, 2009)	2075 (2012, 2137)	129 (125, 132)	6.6 (6.3, 6.9)	2045 (1982, 2108)	99 (96, 102)	5.1 (4.8, 5.3)	30 (29, 30)	1.4 (1.4, 1.5)
Age 4–8y^b^	1684 (1642, 1726)	1772 (1731, 1812)	88 (82, 94)	5.2 (4.8, 5.6)	1754 (1713, 1795)	70 (65, 75)	4.2 (3.8, 4.5)	18 (16, 19)	1.0 (0.9, 1.1)
Age 9–13y^c^	1906 (1839, 1974)	2033 (1970, 2096)	127 (117, 136)	6.6 (6.0, 7.3)	2002 (1938, 2066)	96 (89, 103)	5.0 (4.5, 5.5)	31 (29, 33)	1.5 (1.4, 1.7)
Age 14–18y^d^	2151 (2030, 2273)	2308 (2184, 2433)	157 (151, 162)	7.3 (6.9, 7.6)	2272 (2148, 2396)	120 (116, 125)	5.6 (5.3, 5.9)	36 (35, 37)	1.6 (1.5, 1.7)
Vitamin C (mg)									
Overall	55 (50, 60)	125 (120, 129)	70 (68, 72)	127 (114, 140)	88 (84, 93)	33 (32, 34)	60.2 (54.0, 66.3)	37 (36, 38)	41.8 (39.3, 44.3)
Age 4–8y	50 (41, 59)	96 (88, 104)	45 (42, 48)	90.1 (70.5, 110)	74 (65, 83)	24 (22, 25)	46.7 (36.6, 56.7)	22 (20, 23)	29.6 (24.9, 34.3)
Age 9–13y	52 (45, 59)	123 (116, 129)	70 (66, 75)	135 (112, 158)	84 (78, 91)	32 (30, 34)	61.4 (50.8, 71.9)	38 (36, 41)	45.7 (41.1, 50.3)
Age 14–18y	61 (51, 70)	146 (135, 156)	85 (83, 88)	141 (119, 163)	101 (91, 111)	40 (39, 42)	66.5 (56.0, 76.9)	45 (44, 47)	44.7 (40.5, 48.8)
Dietary fiber (gm)									
Overall	12.7 (12.1, 13.3)	13.6 (13.0, 14.3)	0.9 (0.9, 1.1)	7.5 (6.9, 8.1)	15.9 (15.3, 16.5)	3.2 (3.1, 3.3)	25.0 (23.2, 26.8)	−2.2 (−2.3, −2.1)	−14.1 (−14.9, −13.2)
Age 4–8y	11.1 (10.5, 11.7)	12.0 (11.4, 12.6)	0.9 (0.8, 1)	8.4 (7.3, 9.5)	13.3 (12.8, 13.9)	2.3 (2.1, 2.4)	20.4 (18.5, 22.3)	−1.3 (−1.4, −1.2)	−9.9 (−10.8, −9.1)
Age 9–13y	13.0 (12.0, 14.0)	13.8 (12.8, 14.7)	0.7 (0.7, 0.8)	5.7 (4.9, 6.6)	16.1 (15.2, 16.9)	3.1 (2.8, 3.3)	23.6 (20.5, 26.7)	−2.3 (−2.5, −2.2)	−14.5 (−16, −13)
Age 14–18y	13.5 (12.6, 14.3)	14.6 (13.7, 15.5)	1.1 (1.1, 1.2)	8.5 (7.9, 9.1)	17.3 (16.4, 18.3)	3.9 (3.7, 4)	28.7 (26.9, 30.6)	−2.7 (−2.8, −2.6)	−15.7 (−16.6, −14.9)
Calcium (mg)									
Overall	1043 (988, 1099)	1166 (1112, 1220)	123 (119, 127)	11.8 (10.9, 12.6)	1064 (1009, 1119)	21 (20, 21)	2.0 (1.9, 2.1)	102 (99, 105)	9.6 (8.9, 10.3)
Age 4–8y	1021 (956, 1086)	1097 (1032, 1162)	76 (71, 81)	7.4 (6.7, 8.1)	1036 (971, 1101)	15 (14, 16)	1.4 (1.3, 1.6)	61 (57, 65)	5.9 (5.3, 6.4)
Age 9–13y	1029 (957, 1102)	1156 (1083, 1229)	127 (118, 135)	12.3 (11.1, 13.5)	1049 (977, 1122)	20 (19, 22)	2.0 (1.8, 2.1)	107 (99, 114)	10.2 (9.2, 11.1)
Age 14–18y	1070 (987, 1154)	1221 (1136, 1305)	150 (145, 155)	14.0 (13.0, 15.1)	1096 (1012, 1179)	25 (24, 26)	2.4 (2.2, 2.5)	125 (121, 129)	11.4 (10.6, 12.3)
Potassium (mg)									
Overall	2084 (2004, 2163)	2467 (2389, 2544)	383 (371, 395)	18.4 (17.4, 19.4)	2378 (2300, 2457)	295 (285, 304)	14.1 (13.4, 14.9)	88 (86, 91)	3.7 (3.5, 3.9)
Age 4–8y	1897 (1826, 1968)	2159 (2099, 2220)	262 (244, 280)	13.8 (12.5, 15.1)	2107 (2043, 2170)	209 (195, 224)	11.0 (10.0, 12.1)	53 (49, 56)	2.5 (2.3, 2.7)
Age 9–13y	2002 (1914, 2091)	2379 (2285, 2474)	377 (350, 404)	18.8 (17.3, 20.4)	2287 (2194, 2380)	285 (263, 306)	14.2 (13.0, 15.4)	92 (86, 99)	4.0 (3.8, 4.3)
Age 14–18y	2278 (2129, 2427)	2744 (2592, 2897)	467 (451, 482)	20.5 (19.1, 21.9)	2636 (2484, 2788)	358 (346, 371)	15.7 (14.7, 16.8)	108 (105, 112)	4.1 (3.8, 4.4)
Cost ($/d)									
Overall	4.33 (4.1, 4.56)	4.67 (4.44, 4.90)	0.34 (0.33, 0.35)	7.8 (7.3, 8.3)	4.97 (4.74, 5.21)	0.64 (0.62, 0.66)	14.9 (13.9, 15.9)	−0.31 (−0.32, −0.3)	−6.1 (−6.5, −5.8)
Age 4–8y	3.49 (3.37, 3.6)	3.76 (3.64, 3.88)	0.28 (0.25, 0.3)	7.9 (7.2, 8.6)	3.94 (3.82, 4.06)	0.46 (0.43, 0.49)	13.1 (12.2, 14.1)	−0.18 (−0.2, −0.17)	−4.6 (−5.0, −4.3)
Age 9–13y	4.24 (4.12, 4.35)	4.54 (4.42, 4.66)	0.3 (0.28, 0.33)	7.2 (6.5, 7.9)	4.86 (4.74, 4.97)	0.62 (0.58, 0.67)	14.7 (13.4, 16.0)	−0.32 (−0.34, −0.3)	−6.6 (−7.0, −6.1)
Age 14–18y	4.96 (4.47, 5.45)	5.37 (4.87, 5.87)	0.41 (0.39, 0.43)	8.3 (7.6, 9.0)	5.75 (5.24, 6.25)	0.78 (0.76, 0.81)	15.8 (14.4, 17.2)	−0.37 (−0.39, −0.36)	−6.5 (−7.1, −6.0)

^a^ The mean shortfall in the overall population among those with a fruit shortfall was 1.09 (95 % CI 1.06, 1.12)

^b^ The mean shortfall among those 4–8y with a fruit shortfall was 0.78 (95 % CI 0.72, 0.83)

^c^ The mean shortfall among those 9–13y with a fruit shortfall was 1.05 (95 % CI 0.98, 1.13)

^d^ The mean shortfall among those 14–18y with a fruit shortfall was 1.33 (95 % CI 1.28, 1.37)

As expected, results were stronger when restricted to the child population with a shortfall in total fruit consumption (Table 3), where energy intakes increased by 6.6 % (95 % CI 6.3, 6.9) or 129 kcal (95 % CI 125, 132) and 5.1 % (95 % CI 4.8, 5.3) or 99 kcal (95 % CI 96, 102) in the FJ + WF and WF models, respectively. In the FJ + WF model, vitamin C, dietary fiber, calcium and potassium were increased by +127 % (+70 mg/d), +7.5 % (+0.9 g/d), +11.8 % (+123 mg/d) and +18.4 % (+383 mg/d), respectively. In the WF model, vitamin C, dietary fiber, calcium and potassium were increased by +60 % (+33 mg/d), +25 % (+3.2 g/d), +2.0 % (+21 mg/d) and +14.1 % (+295 mg/d), respectively. Similar to the results from the total population, the FJ + WF model increased vitamin C, potassium and calcium by a greater amount than the WF model, while the WF model increased dietary fiber more than the FJ + WF model. The FJ + WF model increased diet costs by 7.8 % (95 % CI 7.3, 8.3 %), while the WF model increased diet costs by 14.9 % (95 % CI 13.9, 15.9). When comparing the two models to each other with regards to cost, the WF model resulted in 6.1 % (95 % CI 5.8, 6.5) or $0.31/d (95 % CI 0.30, 0.32) higher diet costs Results were generally consistent across age groups (see Table 3) and when stratified by race/ethnicity and family income (data not shown).

## Discussion

The shortfall in fruit consumption by US children can be addressed in a number of ways. One is through increased consumption of whole fruit; the approach currently favored by the new WIC package, professional organizations and childcare/preschool licensors [34]. As shown, this approach would increase dietary intakes of vitamin C, potassium, and dietary fiber by a meaningful amount (i.e., more than a 10 % increase from observed diets). Since most fruits have low energy densities, this increase in dietary nutrient density can be accomplished with only a modest increase in energy intakes. However, when compared to the whole fruit plus fruit juice model, the whole fruit alone model resulted in a significantly higher diet cost, and had smaller gains for potassium, calcium and vitamin C.

While both models increased diet costs, the addition of whole fruit alone increased diet costs by a significantly greater amount than a combination of whole fruit and fruit juice (+$0.21/d [95 % CI 0.20, 0.22] for all children and + 0.31/d [95 % CI 0.30, 0.32] among those with a shortfall in fruit consumption). While higher-income families may be able to more readily absorb higher diet costs, higher costs may pose a larger barrier to lower-income families on limited food budgets. Given previous evidence of a socioeconomic gradient in fruit consumption [4, 6], the provision of fresh fruit may pose a challenge to this population. Further, interventions or policy changes that are not cost-neutral may pose challenges for interventions in institutional settings, including day care centers, pre-schools and schools that need to provide optimal nutrition at an affordable cost [35, 36].

In the FJ + WF model, the fruit shortfall was addressed through a combination of whole fruit and 100 % fruit juice. The amount of juice permitted in modeling for each age group was capped by the AAP guidelines. As shown, the mixed model (whole fruit and juice) increased dietary intakes of vitamin C, potassium, and calcium by a meaningful amount, but fiber was only modestly increased (i.e., less than a 10 % relative change from observed diets). These nutrient gains were accompanied by only a modest increase in energy intakes (+4.5 % for the entire population and +6.6 % for those with a fruit shortfall). Consumption of vitamin C is generally adequate, so increasing vitamin C intake is unlikely to have a benefit to public health [37]. However, increasing potassium, calcium and dietary fiber in the American diet is a priority outlined in the 2010 Dietary Guidelines for Americans [1, 2, 38].

Reaching recommendations using a combination of fruit and juice could be accomplished a much less costly manner than whole fruit alone, though it is important to note that both require an increase in spending. One economically viable option to meet fruit goals is to combine some 100 % fruit juice with whole fruit. Public health interventions to improve the quality of children’s diets need to take nutritional, behavioral and economic factors into account.

In terms of diet costs, both potassium and dietary fiber were previously identified as nutrients associated with increased diet costs [26]. This relationship is driven by the higher energy-adjusted cost of some key sources of dietary fiber and potassium, including vegetables and fruit. In the current study, a combination of fruit juice and whole fruit increased potassium intakes by 14–21 % among children/adolescents consuming too little fruit, while the cost of the diet only increased by 7–8 %. In a previous study, replacement of fruit juice with different types of whole fruit on a serving-per-serving basis did not result in an increase in diet costs for the entire child/adolescent population, though this study did not evaluate the economic impact of meeting fruit guidelines and instead manipulated existing dietary habits [39].

Beyond economic constraints, there are additional challenges in increasing whole fruit consumption among children. One factor that might drive a preference for fruit juice over whole fruit includes the ease of storage, preparation and portioning, which may be particularly important in institutional settings [41]. Spoilage and wastage due to over-ripening of fresh fruit is also a challenge. According to USDA estimates, 25 % of fresh fruit at the consumer level is lost due to over-ripening or spoilage, compared to 11 % for processed fruits (which includes fruit juice along with canned and frozen fruit) [42]. Furthermore, fruit juices may be more convenient for parents and caregivers who have time constraints and may look for easy and quick options [40, 41]. Beyond individual-level barriers, there is some evidence that individuals residing in more deprived neighborhoods may have limited access to fresh fruits at local stores [43], which may influence fresh/whole fruit consumption [44]. In addition, while access to whole/fresh fruit in stores may vary by neighborhood characteristic and type of store, fruit juice is generally available [45].

This study had a number of limitations. First, the consequences of adding whole fruit and fruit juice to diets of children to remove any shortfall in fruit consumption fails to measure the actual dietary behavior of children/adolescents. As such, our findings represent an estimate of the potential dietary and economic impact of adding fruit juice/whole fruit to the diets of children/adolescents. As a related issue, this model often included small amounts of juice (e.g., 1–2 fluid ounces), which would be difficult to consume in a real-world setting, where most children likely consume juice from larger cups or packages. A second limitation is that our models did not explore other potential substitution scenarios that may improve diet quality for children/adolescents. For example, replacing snacks foods or desserts with whole fruit would further reduce total energy intake, increase nutrient density and fruit consumption, while reducing consumption of added sugars, sodium or solid fats. Determining the foods to replace with fruit is a subjective exercise, and may fail to capture probable dietary behaviors. In addition, this analysis did not evaluate the nutritional or economic consequences of altering other components of the diet, such as increasing vegetable or whole grain consumption. Third, the monetary cost of adding whole fruit and fruit juice to the diets of children is based on a single, nationally representative database of prices that does not account for seasonal or regional variations in food prices, or difference in prices between stores and restaurants.

## Conclusions

Increasing fruit consumption among American children and adolescents is an important component of improving diet quality and nutrient adequacy in this population. While both approaches used here to fill the fruit shortfall resulted in beneficial changes to vitamin C, dietary fiber, potassium and calcium consumption, the whole fruit alone model was clearly superior for dietary fiber, but the combined model was superior for the other nutrients, and was able to fill the shortfall in fruit consumption at a significantly lower-cost than through whole fruit alone. Whether whole fruit or a combination of whole fruit and modest amounts of fruit juice are used to address the fruit shortfall, there will be beneficial effects on diet quality and nutrient adequacy. Within cost constraints, a combination of whole fruit and 100 % fruit juice, capped at AAP recommended values, may be a viable option to improve the nutrient adequacy, particularly in terms of vitamin C, potassium and calcium, of diets consumed by American children and adolescents. It is important to note that the combination of fruit juice and whole fruit provided much less dietary fiber than the use of whole fruit alone. In addition, interventions or policies that can increase consumption of whole fruit in as cost-neutral a manner as possible should also be considered. Such strategies may include subsidies or increased marketing of whole fruit, as well as development of novel and convenient packaged or pre-sliced fruits, which may be more readily incorporated in school or childcare settings.

## Abbreviations

AAP, American Academy of Pediatrics; CNPP, Center for Nutrition Policy and Promotion; MPED, MyPyramid Equivalents Database; NHANES, National Health and Nutrition Examination Survey; QFAHPD, Quarterly Food At-Home Price Database; USDA, United States Department of Agriculture; WIC, Women, Infants and Children program
